# Profiling of Ubiquitination Pathway Genes in Peripheral Cells from Patients with Frontotemporal Dementia due to *C9ORF72* and *GRN* Mutations

**DOI:** 10.3390/ijms16011385

**Published:** 2015-01-08

**Authors:** Maria Serpente, Chiara Fenoglio, Sara M. G. Cioffi, Rossana Bonsi, Andrea Arighi, Giorgio G. Fumagalli, Laura Ghezzi, Elio Scarpini, Daniela Galimberti

**Affiliations:** Neurology Unit, Department of Pathophysiology and Transplantation, “Dino Ferrari” Center, University of Milan, Fondazione Ca’ Granda, IRCCS Ospedale Maggiore Policlinico, Milan 20122, Italy; E-Mails: chiara.fenoglio@unimi.it (C.F.); sara.cioffi@unimi.it (S.M.G.C.); rossana.bonsi@unimi.it (R.B.); andrea.arighi@yahoo.it (A.A.); giorgiofumagalli@hotmail.com (G.G.F.); lauraghezzi@me.com (L.G.); elio.scarpini@unimi.it (E.S.); daniela.galimberti@unimi.it (D.G.)

**Keywords:** frontotemporal lobar degeneration, progranulin, *C9ORF72*, ubiquitination, E2 ubiquitin conjugating enzyme

## Abstract

We analysed the expression levels of 84 key genes involved in the regulated degradation of cellular protein by the ubiquitin-proteasome system in peripheral cells from patients with frontotemporal dementia (FTD) due to *C9ORF72* and *GRN* mutations, as compared with sporadic FTD and age-matched controls. A SABiosciences PCR array was used to investigate the transcription profile in a discovery population consisting of six patients each in *C9ORF72*, *GRN*, sporadic FTD and age-matched control groups. A generalized down-regulation of gene expression compared with controls was observed in *C9ORF72* expansion carriers and sporadic FTD patients. In particular, in both groups, four genes, *UBE2I*, *UBE2Q1*, *UBE2E1* and *UBE2N*, were down-regulated at a statistically significant (*p* < 0.05) level. All of them encode for members of the E2 ubiquitin-conjugating enzyme family. In *GRN* mutation carriers, no statistically significant deregulation of ubiquitination pathway genes was observed, except for the *UBE2Z* gene, which displays E2 ubiquitin conjugating enzyme activity, and was found to be statistically significant up-regulated (*p* = 0.006). These preliminary results suggest that the proteasomal degradation pathway plays a role in the pathogenesis of FTD associated with TDP-43 pathology, although different proteins are altered in carriers of *GRN* mutations as compared with carriers of the *C9ORF72* expansion.

## 1. Introduction

Frontotemporal lobar degeneration (FTLD) is a group of complex disorders resulting from the progressive deterioration of the frontal and anterior temporal lobes of the brain. The subcategories of the disease are defined by their dominant clinical symptom in patients; the behavioral variant frontotemporal dementia (FTD) that accounts for two-thirds of patients, and two language variants classified on their effects on fluency (Progressive non-fluent aphasia, PNFA) or semantic difficulties in communicative speech and understanding the semantic content of language (Semantic dementia, SD). In addition, FTD may overlap clinically with motor neuron disease (FTD-MND) [1]. Several genes have been identified as causative of autosomal dominant FTLD. In particular, microtubule associated protein tau (*MAPT*), progranulin (*GRN*) and chromosome 9 open reading frame (*C9ORF*)*72* are considered the most important players of FTD, responsible for the majority of inherited cases. Mutations in *GRN* account for 5% to 11% of cases [2,3]. Upon autopsy, *GRN* mutation carriers exhibit cerebral atrophy and show a highly constituent pattern corresponding to FTLD-TAR DNA binding protein (TDP)-43 type A, which is characterized by numerous short dystrophic neurites and neuronal cytoplasmatic inclusions [4]. In late 2011, a repeated hexanucleotide in the first intron of *C9ORF72* was found to be the most common genetic cause of amyotrophic lateral sclerosis (ALS) and FTD, with or without MND [5,6]. This expansion accounts for about 6% of cases [7], and is categorized histologically as a Type B pathology, with inclusion bodies in neurons and in glial cells that are TDP-43 positive [8]. However, Al-Sarraj and colleagues, described a subset of TDP-43 proteinopathy patients, carrying *C9ORF72* expansion, who had unusual and abundant p62 positive, but TDP-43 negative inclusions in the cerebellum and hippocampus [9].

Efficient and rapid elimination of misfolded proteins is critical to the maintenance of cellular health. Under normal conditions, protein homeostasis is achieved through a molecular pathway called the ubiquitin-proteasome system (UPS) [10]. The UPS is a highly coordinated system, which regulates the degradation of a wide variety of proteins. Therefore, it has been supposed that dysfunction of the UPS is implicated in the pathogenesis of several neurodegenerative diseases, such as Huntington’s disease (HD) and Alzheimer’s disease (AD) [11]. Studies in animal models indicate that early impairment of the UPS could be considered the primary mediator of neurodegeneration, raising the possibility of proteostasis-based therapies to slow disease progression [12,13]. Therefore, the UPS represents, for mammalian cells, a major defense against misfolded proteins, particularly in post-mitotic neurons. In healthy conditions, proteins are marked for proteosomal degradation by covalent conjugation of ubiquitin (Ub), a highly conserved protein composed by 76 amino acids, in a three-step cascade. Firstly, the ubiquitin-activating enzyme (E1) activates Ub in an ATP-dependent reaction. Following activation, Ub is transferred in a second thioester linkage to one of several ubiquitn-conjugating enzymes (E2) [14]. A third class of enzyme, the ubiquitin ligases (E3s), mediates the attachment of poly-ubiquitin chains to specific substrate proteins. Poly-ubiquitinated proteins are recognized and subsequently degraded by the 26S proteasome [15]. Similar to other posttranslational modifications, the process of ubiquitination is reversible under the influence of specific de-ubiquitinating enzymes (DUBs) [16]. Both E2 and E3 proteins exist as large families and different combinations of E2s with different E3 proteins define the substrate specificity.

Another mechanism to remove misfolded protein is autophagy, the main cellular catabolic route for protein aggregates and damaged organelles. Autophagy plays a critical role in cytoprotection by preventing the accumulation of toxic proteins and acting in various aspects of immunity, including the elimination of invading microbes and its participation in antigen presentation. In the last decade, emerging evidence revealed that autophagy can distinguish and direct specific cargos to the lysosome.

Protein degradations performed by the UPS and autophagy were regarded for a long time as complementary but separate mechanisms [17]. However, on the basis of recent studies, there are overlaps between them. The way of degradation of a misfolded, redundant, or unneeded protein may be often governed by the momentary activity or capacity of these systems or, in some cases, determined by strict regulation. Moreover, the two pathways use common adaptors capable of directing ubiquitinylated target proteins to both. For example, recognition of ubiquitinylated proteins during autophagy is mediated by ubiquitin receptors interacting with ubiquitin noncovalently, via their ubiquitin-binding domains. Sequestosome 1 (p62/SQSTM1), the first protein reported to have such an adaptor function [18], was originally discovered as a scaffold in signaling pathways regulating cell growth and proliferation.

Recently, several rare mutations in the *SQSTM1* gene, were reported in patients with FTLD and ALS [18,19,20]. Moreover, van der Zee *et al.*, identified additional variants in *SQSTM1* in FTLD patients [21].

Given the importance of UPS impairment in the pathogenesis of neurodegenerative diseases, in the present study we aimed at determining the contribution of ubiquitination pathway gene expression to the pathogenesis of FTLD. In particular, we analyzed the expression profile of 84 key genes involved in the UPS in peripheral cells from *C9ORF72* and *GRN* mutation carriers, who are likely characterized by TDP-43 deposition in the brain, as compared with sporadic FTLD and age-matched controls.

## 2. Results

**Figure 1:**
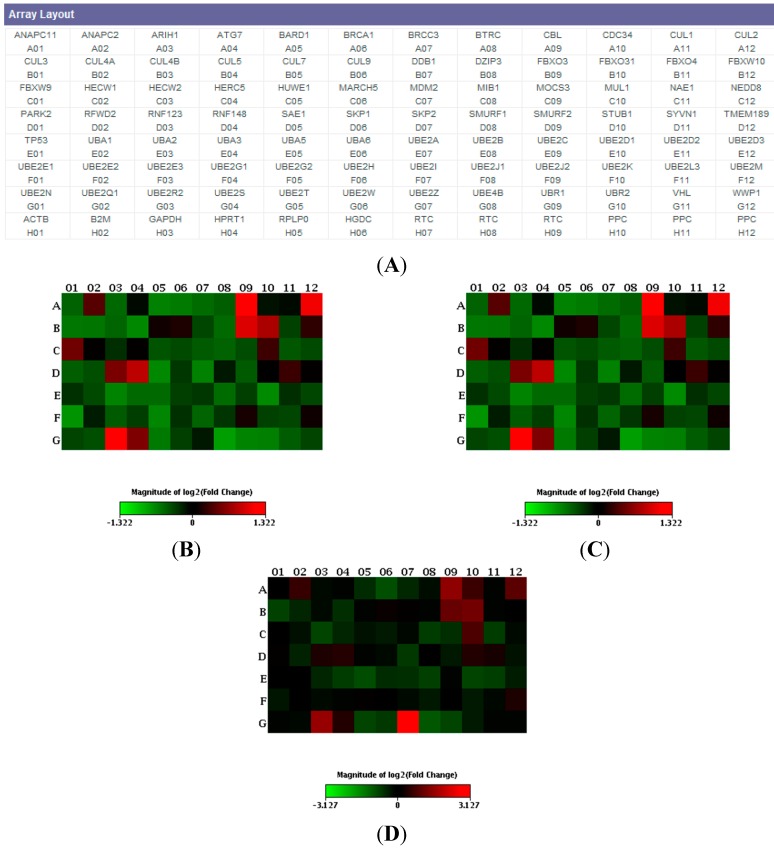
(**A**) Layout of the ubiquitination (ubiquitylation) pathway PCR array (PAHS-079Z). Heat maps of patients *versus* controls; (**B**) *C9ORF72*; (**C**) sporadic; and (**D**) *GRN*. Data are expressed as fold change (fold difference) and each square represents a gene of the ubiquitination pathway. Green indicates down-regulation, red up-regulation.

Gene expression profiles of 84 key genes involved in the regulated degradation of cellular proteins by the ubiquitin-proteasome system (Figure 1A) were performed in PBMCs from six each of: sporadic FTD patients, *C9ORF72* expansion carriers, *GRN* Thr272fs (g.1977_1980delCACT) carriers, and age-matched healthy control groups.

A generalized down-regulation of the gene expression profile in *C9ORF72* expansion carriers and sporadic bvFTD patients compared with controls was observed (Figure 1B,C). In particular, in both groups, four genes were found statistically significant down-regulated (mean ± SD *versus* controls): *UBE2N* (sporadic FTD patients: −1.54 ± 1.46 fold decrease, *p* = 0.014; *C9ORF72* expansion carriers: −1.2 ± 1.24 fold decrease, *p* = 0.011), *UBE2Q1* (sporadic FTD patients: −1.48 ± 1.32 fold decrease, *p* = 0.014; *C9ORF72* expansion carriers: −1.13 ± 1.12 fold decrease, *p* = 0.0027), *UBE2E1* (sporadic patients: −1.94 ± 1.27 fold decrease, *p* = 0.002; *C9ORF72* expansion carriers: −1.69 ± 1.47 fold decrease, *p* = 0.007), *UBE2I* (sporadic FTD patients: −1.57 ± 1.32 fold decrease, *p* = 0.013; *C9ORF72* expansion carriers: −1.42 ± 1.30 fold decrease, *p* = 0.011). All four genes encode for members of the E2 ubiquitin-conjugating enzyme family.

In *GRN* mutation carriers, no statistically significant deregulation of ubiquitination pathway genes was observed, except for the *UBE2Z* gene, which was found to be statistically significant up-regulated (8.73 ± 1.06 fold increase over controls, *p* = 0.006, Figure 1D). Similarly to the previous four genes described, *UBE2Z* gene displays E2 ubiquitin conjugating enzyme activity.

In addition, despite that the significant threshold was not reached, the following genes were up-regulated in sporadic, *C9ORF72*, and *GRN* patients *versus* controls, independent of the presence of mutations: *CBL* (2.79 ± 1.3, 2.5 ± 1.8, 2.81 ± 0.8 fold increase, respectively, *p* > 0.05), *FBXO3* (2.48 ± 1.4, 1.97 ± 1.08, 2.10 ± 0.8, respectively, *p* > 0.05) and *UBE2R2* (2.81 ± 1.4, 2.35 ± 1.7, 2.98 ± 0.78, respectively, *p* > 0.05).

## 3. Discussion

Here, we report that genes involved in the regulated degradation of cellular proteins by the ubiquitin-proteasome system are deregulated in FTD. In particular, specific profiles have been found in carriers of *GRN* and *C9ORF72* mutations, which are associated with TDP-43 pathology. We thus could hypothesize that different genetic defects (*GRN* or *C9ORF72* mutations) converge, at some point during neurodegeneration, on the same pathway.

The control of protein synthesis and turnover is essential for the health of all cells. In neurons this equilibrium takes on the additional importance of supporting and regulating the highly dynamic connections between neurons, which are necessary for cognitive functions. The UPS is able to address this balance thanks to the combined activity of over 500 enzymes working together to regulate protein-protein interaction and eliminate unwanted proteins. Evidence continues to mount for the necessity of UPS involvement in the dynamic remodelling of synaptic structures following synaptic activity [22,23], but the full role of ubiquitination pertaining to synaptic structure remains incompletely understood [24]. Improper clearance of proteins is believed to be a causative or contributing factor in many neurodegenerative diseases, which are often characterized by the accumulation of aggregated proteins [25]. In particular, UPS dysfunction has been reported in the most common neurodegenerative diseases including AD, HD and Parkinson’s Disease (PD), but little is known about the role of ubiquitination in the pathogenesis of FTLD.

Our results reveal a generalized down-regulation of ubiquitination gene expression in sporadic patients and *C9ORF72* expansion carriers. In particular, four genes *UBE2I*, *UBE2Q1*, *UBE2E* and *UBE2N* were down-regulated at statistically significant levels in both groups. On the contrary, no statistically significant deregulation of the ubiquitination gene pathway was observed in *GRN* mutation carriers, except for the *UBE2Z* gene, which was significantly up-regulated in *GRN* mutation carriers as compared with controls. Conversely, a few genes showed a trend towards an up-regulation all patients, independent of the presence of mutations, including *CBL*, *FBXO3* and *UBE2R2.* However, to date, these genes are not very well characterized, despite being implicated in the ubiquitin ligase activity.

*UBE2I*, *UBE2Q1*, *UBE2E* and *UBE2N* genes encodes for members of the E2 ubiquitin-conjugating enzyme family, but little is known about the function of this enzyme. Recently, UBE2I protein was associated with early events occurring in astrocytes from animal models of amyotrophic lateral sclerosis [26]. Moreover, the expression levels of UBE2Q1 were found down regulated in rat brain following traumatic brain injury [27]. Hans *et al.*, demonstrated that UBE2E protein binds TDP-43 directly, and identified a new potential modifier of TDP-43 neurotoxiticy [28]. Taken together these findings show that a modulation of ubiquitination enzymes might become useful in future preclinical studies, but the complete mechanism of TDP-43 turnover remains to be identified.

In addition, it is interesting to note that the *UBE2Z* gene is located on chromosome 17q21, quite close to the *GRN* gene position and is widely expressed in human tissues [29]. According to Hapmap data, they are not however in the same linkage disequilibrium block, suggesting that mutations in *GRN* gene should not influence *UBE2Z* genomic structure and transcription.

Regarding the population studied, cases were homogeneous in terms of clinical presentation and phenotype and age at onset. Nevertheless, we acknowledge that our cohort is quite small and no neuropathological confirmation is available. This point is important especially for sporadic patients, because we cannot predict the pathology (TDP-43 *versus* tau). Therefore a replication in a larger and pathologically confirmed population is advisable. On the other hand, although preliminary, data we obtained from samples deriving from patients may help to better understand processes occurring *in vivo*, which may hopefully result in the identification of potential therapeutic targets.

Lastly, the UPS is not alone in its handling of unwanted proteins. The autophagy system is the other major mechanism by which protein clearance is achieved and it may be especially important given the p62 pathology in *C9ORF72* cases. Given that, this kind of analysis should be conducted also for autophagy gene expression pathway.

## 4. Materials and Methods

### 4.1. Patients and Controls

Eighteen patients with FTD (six each of *GRN* mutation carriers, *C9ORF72* expansion carriers and the sporadic groups) were recruited at the Alzheimer Unit of the Fondazione Cà Granda, IRCCS Ospedale Maggiore Policlinico, University of Milan (Milan, Italy). All patients underwent a standard battery of examinations, including medical history, physical and neurological examination, screening laboratory tests, neurocognitive evaluation and imaging. Cognitive dysfunctions were assessed by the clinical dementia rating (CDR), the mini mental state examination (MMSE), the frontal assessment battery (FAB), the Wisconsin Card Sorting Test (WCST), and the Tower of London test. The presence of significant vascular brain damage was excluded (Hachinski Ischemic Score < 4). The diagnosis of FTD was made according to current consensus criteria [30] and subsequent revisions [31,32].

The control group consisted of six non-demented volunteers matched for ethnic background and age, without memory and psycho-behavioural dysfunctions (MMSE ≥ 28). Informed consent to participate in this study was given by all subjects or their caregivers. Characteristics of patients and controls are summarized in Table 1.

**Table 1:** Characteristics of frontotemporal dementia (FTD) patients and controls.

Characteristics	Sporadic	*C9ORF72*	*GRN*	Controls
Number of patients	6	6	6	6
Gender (M:F)	4:2	5:1	4:2	3:3
Mean age (years ± SEM)	66.3 ± 8.6	63.9 ± 8.1	62 ± 9.4	65 ± 7.3
Mean Age at onset (years ± SEM)	64.5 ± 0.44	61 ± 0.36	59 ± 0.98	–

SEM = Standard error of mean.

### 4.2. Screening of GRN and C9ORF72 Mutations

High molecular weight DNA was isolated from whole blood using a Flexigene Kit (Qiagen, Hildren, Gemany). *GRN* sequencing was performed by direct sequencing, as previously described [33]. *C9ORF72* genotyping was carried out by repeat-primed polymerase chain reaction and sequencing [5]. This method allows detection of 30 to 50 repeats; According to current literature [34], a characteristic stutter amplification pattern (>40 repeats) on the electropherogram is considered evidence of a pathogenic repeat expansion.

### 4.3. Total mRNA Isolation from Peripheral Blood Mononuclear Cells (PBMC)

For each subject, 14 mL of blood have been collected in two BD VacutainerR CPTTM (1 mL NC, 2 mL Ficoll) as previously described [35]. From each tube, PBMCs were separated by gradient centrifugation and total RNA extracted with the single step acid phenol method, using Trizol (Invitrogen, Carlsbad, CA, USA). RNA purity was measured by optical density and only samples with an OD 260/280 ratio ranging from 1.8 to 2 and an OD 260/230 of 1.8 or greater were used.

### 4.4. Screening of Ubiquitination Pathways by PCR Array

RNA was retrotranscribed with RT2 First Strand Kit (SABiosciences, Frederick, MD, USA), according to the instruction of the manufacturer. For real time PCR experiments, the ubiquitination (ubiquitylation) pathway PCR array (PAHS-079Z, Figure 1) was used and runs were performed in an Applied BioSystems StepOne Plus system (Foster City, CA, USA). The array profiles the expression of 84 key genes involved in the regulated degradation of cellular proteins by the ubiquitin-proteasome system. These arrays included also five housekeeping genes (*ACTB*, *B2M*, *G3PDH*, *HPRT1* and *RPLP0*) for the proper normalization of the data, mRNA reverse transcription control and a positive PCR control (Figure 1A).

### 4.5. Statistical Analysis

The SABiosciences PCR Array data analysis was based on ΔΔ*C*_t_ method with normalization of the raw data to housekeeping genes (using the software available at http://www.sabiosciences.com/pcarraydataanalysis.php). *p* Values were calculated based on a Student’s *t*-test of the replicate 2^−Δ*C*t^) values for each gene in the control and FTD groups. Best hits were chosen based on statistical significance (*p* < 0.05). Haploview 4.2 software was used to test for linkage disequilibrium (LD) blocks.

## 5. Conclusions

In conclusion, knowledge of the UPS components that are involved in FTD pathogenesis will be helpful for the identification of potential therapeutic targets, which aim to limit, at early stages, the accumulation of misfolded proteins without disturbance of this major proteolytic pathway.
